# Epstein‐Barr Virus Transformed B Cells From Systemic Lupus Erythematosus and Multiple Sclerosis Patients Differ in EBV Lytic and Latency Marker Expression

**DOI:** 10.1002/iid3.70411

**Published:** 2026-03-23

**Authors:** Ali Afrasiabi, Wade J. Bocking, Stephen D. Schibeci, Samantha P. L. Law, Nicole L. Fewings, Ming‐Wei Lin, Stephen J. Sawcer, Maria Ban, Sanjay Swaminathan, Grant P. Parnell

**Affiliations:** ^1^ EBV Molecular Lab, Centre for Immunology and Allergy Research, Westmead Institute for Medical Research University of Sydney Sydney New South Wales Australia; ^2^ Children's Cancer Institute, Lowy Cancer Research Centre UNSW Sydney Sydney New South Wales Australia; ^3^ School of Clinical Medicine, UNSW Medicine & Health UNSW Sydney Sydney New South Wales Australia; ^4^ COVID‐19 Research Lab, Centre for Virology Research, Westmead Institute for Medical Research University of Sydney Sydney New South Wales Australia; ^5^ Department of Clinical Immunology and Allergy Westmead Hospital and Institute of Clinical Pathology & Medical Research (ICPMR) Westmead New South Wales Australia; ^6^ Department of Clinical Neurosciences University of Cambridge Cambridge UK; ^7^ School of Medical Sciences, Faculty of Medicine and Health The University of Sydney Sydney New South Wales Australia

**Keywords:** EBV, latent, LCL, lytic, MS, SLE

## Abstract

Epstein‐Barr virus (EBV) is an important environmental risk factor in the development of several autoimmune conditions, with the mechanisms still to be fully elucidated. EBV primarily infects memory B cells, transitioning between lytic (active) and latent (dormant) phases of infection. Our group has previously proposed two molecular mechanisms linking EBV pathogenesis to autoimmunity: one indicates that EBV lytic switching contributes to systemic lupus erythematosus (SLE) pathogenesis, while another posits that latency III is more crucial in the development of multiple sclerosis (MS). In this study, we tested the proposed molecular model using a cohort of EBV‐transformed lymphoblastoid cell lines derived from individuals with either SLE, MS or healthy controls. Measuring the expression levels of a panel of EBV genes, representing the different phases of the EBV lifecycle, we found compelling proof‐of‐concept evidence validating our proposed model. This discovery highlights promising signatures for further investigation, where the same approach can be explored across other EBV‐associated immune conditions, deepening our understanding of the virus's lifecycle dysregulation in autoimmunity etiology and ultimately aiding in the design of new treatments.

## Graphical abstract

In this study, we examined a small cohort of EBV‐transformed B cells from blood samples of individuals with SLE, MS, or healthy controls, measuring the expression levels of various EBV genes representing different phases of the EBV lifecycle. Our results support our proposed molecular models, demonstrating that EBV lytic phase reactivation is linked to the pathogenesis of SLE, while predominantly remaining in the latent phase in MS. This discovery could lead to novel therapeutic strategies aimed at EBV infection, thereby broadening the treatment options for both SLE and MS.

## 1 Introduction

Epstein‐Barr virus (EBV) is a ubiquitous pathogen affecting approximately 90% of adults [1]. EBV primarily infects B cells and has a dual‐phase lifecycle that encompasses both lytic (active) and latent (dormant) phases [2]. EBV is an important environmental risk factor in developing a range of diverse autoimmune diseases [3], particularly in the context of multiple sclerosis (MS) [4] and systemic lupus erythematosus (SLE) [5].

Previously, we have found genomic and transcriptomic evidence supporting a molecular model suggesting that in MS, EBV has a greater resistance to the switching toward the lytic phase of the virus's life cycle [6], while in SLE, the virus is more geared toward the lytic phase [7]. Understanding the molecular mechanisms that lead EBV to cause diverse autoimmune diseases such as MS and SLE may open new opportunities for future therapeutic targets [8, 9].

To evaluate our proposed molecular models, we generated a cohort of EBV‐transformed B cells, known as lymphoblastoid cell lines (LCLs), derived from blood samples of individuals with either SLE, MS, or Healthy Controls (HC). We then measured the gene expression level of five key markers of the EBV lifecycle, *EBNA1, EBNA2, LMP1, BZLF1*, and *BRLF1* [10] in these LCLs. *EBNA2* is the master regulator of latency III, instrumental in establishing the EBV latency program and inducing immortalization of infected cells [10]. *BZLF1 and BRLF1* are immediate‐early lytic genes [10]. In addition, we measured *EBNA1*, a master regulator expressed throughout all stages of the EBV lifecycle, and LMP1, a latency marker that has also been shown to be upregulated prior to lytic reactivation [10, 11, 12]. Individually, these EBV markers have been previously investigated for their potential roles in SLE and MS pathogenesis [13, 14]. We also tested for co‐expression of these genes within each group and examined for interactions between infected cell phenotypes such as proliferation rate, EBV‐encoded protein expression, and EBV DNA copy number. This study ultimately aimed to provide additional insights into the balance of lytic and latent EBV lifecycle markers within the contexts of MS and SLE.

## 2 Methods

In brief, LCLs derived from both HC individuals and SLE patients were generated from patient PBMCs using EBV‐containing B95‐8 supernatant as previously described [6]. The MS patient‐derived LCLs were generated in the UK using the same EBV strain and were transported to Australia for analysis alongside the HC and SLE samples. The general and clinical characteristics of the LCL samples used in this study are detailed in Table S1. This study received ethics approval from the Western Sydney Local Health District Human Research Ethics Committee, and informed consent was obtained from all participants.

A minimum of two LCLs from each group (HC, MS, SLE LCLs) were thawed and recovered at a time for investigation. Once recovered, mycoplasma negativity was ensured, and cultures were maintained with media (RPMI‐1640 (Sigma‐Aldrich, Cat.# R0883) with 10% FBS (Gibco, Cat.# 10099141)) changes twice weekly. Approximately 1.6 × 10^6^ cells were harvested from LCL cultures grown to a density of 5 × 10^5^ cells/mL for RNA isolation, DNA isolation, and proliferation rate assay. LCLs were then placed back into cryo‐storage using 10% DMSO with 50% FBS in RPMI‐1640 media. cDNA synthesized from isolated RNA was used for RT‐qPCR of the selected EBV life cycle markers using the primer sequences listed in Table S2. Isolated DNA was used to quantify the relative EBV DNA copy number by RT‐qPCR with the primer sequences listed in Table S3. Lastly, cells were used for the determination of LCL proliferation rate, calculated over 7 days with cell counts obtained at 0 and 120 h. The cryopreserved LCLs were subsequently revived and cultured to a density of 5 × 10^5^ cells/mL for quantification of EBNA1, EBNA2, LMP1, and BZLF1 protein levels using flow cytometry. Full methods are available in Supporting Information S1.

## 3 Results

**Figure 1:**
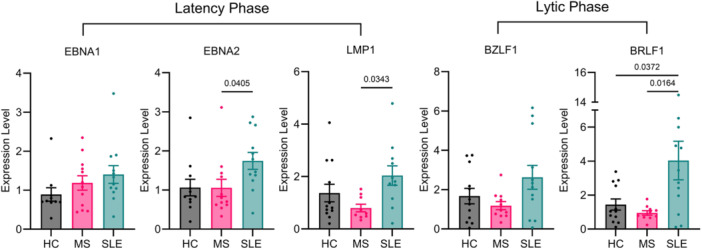
Bar plots visualize expression levels for the EBV genes *EBNA1*, *EBNA2*, *LMP1*, *BZLF1*, and *BRLF1* across HC (*n* = 12), MS (*n* = 12), and SLE (*n* = 12) groups obtained via RT‐qPCR. Expression levels were calculated using the 2^−ΔΔCt^ method against the housekeeping gene *RPL30* and the average expression of the HC groups. Mean expression is denoted by bars with individual data points superimposed and error bars representing ± standard error of the mean. The *p* value for pairwise comparisons is provided for statistically significant differences (*p* < 0.05) between groups.

Investigating the expression of EBV latent and lytic markers at both the gene and protein levels illuminated differences in the regulation of the virus's life cycle between HC LCLs and the MS and SLE LCL groups. The lytic marker *BRLF1*, demonstrated differences in expression across the LCL groups (Figure 1). Specifically, elevated expression in the SLE LCLs was observed when compared to both HC (*p* value: 0.037) and MS (*p* value: 0.016) LCL groups. Furthermore, *EBNA2* and *LMP1 e*xhibited significant differences across the HC, SLE, and MS LCL groups, with notably higher expression of *LMP1* in the SLE compared to the MS LCL groups (*p* value: 0.034). Although there were no statistically significant variations in *EBNA1* and *BZLF1* (Figure 1), trending differences were observed. Empirical *p* values calculated by permutation tests for each pairwise comparison were in agreement with the Tukey HSD test *p* values (Table S4). Furthermore, protein‐level measurements aligned closely with the gene‐level observations for EBNA1, LMP1, and BZLF1 (Figure S1A,B).

Of note, in the SLE LCL group, the mean expression level of all EBV genes was on average 1.81 times higher than the HC LCL group and 2.37 times higher than the MS LCL group. To assess if this was driven by an increased proliferation rate or EBV load in the SLE LCLs, we compared the EBV DNA copy number and proliferation rate across the SLE, MS, and HC LCL cohorts (Figure S2). Notably, the SLE LCLs did not exhibit a higher EBV DNA copy number or proliferation rate compared to the MS or HC LCLs (Figure S2). Rather, the only difference observed was an elevated EBV DNA copy number in the MS LCLs compared to HC (Figure S2A, *p* value: 0.026).

**Figure 2:**
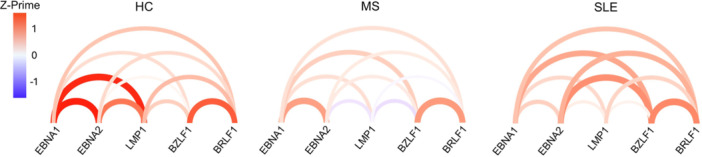
Arc diagrams that illustrate the correlation between EBV genes—*EBNA1, EBNA2, LMP1, BZLF1*, and *BRLF1*—based on their expression levels within HC (*n* = 12), MS (*n* = 12), and SLE (*n* = 12) LCL groups. A red color indicates a positive correlation, while blue signifies a negative correlation. The intensity of the color corresponds to the magnitude of the Z‐prime score, a standardized metric derived from Pearson's correlation coefficients.

The relationship between expression levels of the EBV life cycle genes assayed is presented in Figure 2. The arc diagram for the HC LCL group illustrates distinct and strong interactions within both latent and lytic phases, with pronounced correlation within the latent phase. In contrast, the MS LCL group's arc diagram suggested an increased negative influence from the latent markers on the lytic markers, which may signify a cellular resistance to the switch from latent to lytic phase. Meanwhile, the SLE LCL group displayed a more dispersed pattern of regulatory interactions for all genes, indicating a dysregulation of gene expression control. Notably, the latent phase exhibited weaker correlations compared to the lytic phase. Interestingly, the strong positive correlation observed between *EBNA2* and *BZLF1* seen in the SLE LCL cohort was not observed in either the MS or HC LCLs (Figures 2, S3A–C). The statistical values used for plotting the arc diagrams are provided in Table S5. Further investigation of this correlation showed no significant interaction for either *EBNA2* or *BZLF1* expression levels with both LCL proliferation rate and EBV DNA copy number in the SLE LCL cohort (Figure S3D–G). Exploratory correlation analyses did not identify any significant interactions between clinical disease activity scores and EBV marker expression levels in the MS or SLE LCL cohorts.

## 4 Discussion

Here, we present in vitro evidence suggesting that interactions between EBNA2 and previously established host factors [6, 7, 8, 9] may be responsible for the dysregulation of the EBV lifecycle, likely tied to the timing of the latent‐to‐lytic switch, potentiating the development of autoimmune disease. Across all EBV markers examined, it was evident that the expression of both EBV genes and proteins varied in a context‐dependent manner, whether that is in SLE, MS, or HC LCL groups. Notably, the overall expression levels of the EBV life cycle markers were elevated in the SLE LCL group compared to the MS and HC LCL groups. This increase is unlikely to be driven by proliferation rate or EBV load (Figures S2, S3D–G) but potentially by an enhanced lytic activation or pre‐activation state in the context of SLE, setting the stage for the transition to the lytic phase.

SLE LCLs demonstrated an upregulation in expression of *BRLF1* and *LMP1* when compared to MS LCLs. *BRLF1* upregulation denotes immediate‐early lytic reactivation [10, 13], and *LMP1* is known to be upregulated prior to the lytic phase [10, 11, 12]. Furthermore, the SLE LCL group also exhibited dysregulated gene expression for the latent phase markers, with an emphasis on lytic phase activation, as indicated by the positive correlation between *EBNA2* and *BZLF1*. This correlation was not observed in the other LCL groups (Figures 2 and S3A–C). Taken together, this supports our notion that EBV has a greater propensity to switch toward the lytic phase in the context of SLE [7]. Conversely, the MS LCL group demonstrates a potential resistance to transitioning to the lytic phase, suggested by negative correlations between *EBNA2* and *LMP1* on *BZLF1* and *BRLF1*, respectively, alluding to a preservation of the latency phase in MS (Figure 2).

The notion of an increase in EBV lytic switching in the context of SLE is also supported in previous work by others. Studies involving SLE patient blood samples observed an increase in EBV lytic reactivation markers in patients experiencing disease flares [15, 16]. Furthermore, the upregulated expression of *LMP1* in the SLE LCL cohort observed in this study aligns with previous studies, which suggested that *LMP1* may have a role in the development and maintenance of autoreactive B cells and autoantigens known to enhance pathogenesis [11, 17, 18]. Together, this evidence gives further support for targeting the virus's lytic phase as an alternative therapeutic strategy for SLE patients, particularly those refractory to current treatments. This study also supports the notion of MS‐patient‐derived LCLs having a greater tendency to remain in the latency phase [6]. In addition, a previous study interrogating Cerebral Spinal Fluid and PBMCs of MS patients found evidence of viral latency activation, further reinforcing that the pattern observed in LCLs may be representative of EBV infection in vivo [19]. Taken together, this evidence suggests that targeting the latency phase of EBV in the context of MS may hold greater therapeutic potential. However, for both disease types, it is vital that the association between viral lifecycle and autoimmune pathogenesis is further elucidated, particularly beyond the in vitro setting, such that effective therapeutic avenues can be pursued.

These conclusions are drawn with the caveat of a modest sample size and will require replication with more refined experimental parameters and a larger cohort for validation. Of note in our results, whilst protein expression differences between groups were relatively similar to those seen in the gene expression data (Figure S1A,B), there were some notable differences, including for EBNA2, which may be attributed to post‐translational modifications [20] or methodological factors, including cell passage number or antibody sensitivity. With a larger cohort, confounding factors such as age, sex, and geographical region could be more acutely interrogated. Furthermore, the impacts of treatment, including the type of drug, dosage, duration, and adherence, will also need to be accounted for. All these factors may have had confounding effects on the results of this study, highlighting the need for future experiments to address these issues and validate the results. Increased sample size will also allow for interrogation of EBV in varying disease phenotypes, such as active flares [21], which may further elucidate a role of EBV in progression and severity. In addition, studies that investigate the composition of the B cell compartment in the autoimmune context and how this translates to the EBV‐transformed B cell lines may also give key insights into the interplay between EBV and host B cells.

Pivoting from host factors, future studies may also incorporate spontaneous LCLs, which utilize endogenous EBV for immortalization, as seen in recent studies [21, 22]. This would enable the interrogation of the genomic differences in EBV strains among infected individuals, which may provide additional insights into the role of the virus in autoimmune disease and how EBV strain differences may influence host and virus interactions. In addition, future studies may benefit from incorporating host genetics to elucidate the likely influence of host genetics on the response to EBV infection and its role in autoimmune disease pathogenesis. Furthermore, employing methods such as siRNA transfection to look more closely at specific host and viral factors that may contribute to disease manifestation in response to EBV infection would also be beneficial.

Although limited by a modest sample size and a heterogeneous cohort, this study provides further support for a molecular model of the role of EBV in SLE and MS pathogenesis, where interactions between EBV and host factors may lead to a dysregulation of the EBV lifecycle and potentially contribute to the development of autoimmune disease [6, 7]. Our findings contribute to growing evidence that targeting the EBV life cycle in MS and SLE patients may offer therapeutic benefits, an approach that may also provide novel therapeutic alternatives in other EBV‐associated immune conditions.

## Author Contributions

**Ali Afrasiabi:** formal analysis, methodology, writing – original draft, writing – review and editing. **Wade J. Bocking:** formal analysis, investigation, methodology, writing – original draft, writing – review and editing. **Stephen D. Schibeci:** methodology, supervision, writing – review and editing. **Samantha P. L. Law:** methodology, writing – review and editing. **Nicole L. Fewings:** methodology, writing – review and editing. **Ming‐Wei Lin:** resources, writing – review and editing. **Stephen J. Sawcer:** resources, writing – review and editing. **Maria Ban:** resources, writing – review and editing. **Sanjay Swaminathan:** conceptualization, resources, supervision, writing – review and editing. **Grant P. Parnell:** conceptualization, formal analysis, methodology, resources, supervision, writing – review and editing.

## Conflicts of Interest

The authors declare no conflicts of interest.

## Supporting information


**Supporting Figure S1A**: Protein expression level of the key EBV encoded proteins EBNA1 (FITC), EBNA2 (PE‐Cy5), LMP1 (APC), BZLF1 (PE) compared to the respective gene expression level for HC (n=12), MS (n=12) and SLE (n=12) LCL groups. **Supporting Figure S1B:** Frequency of HC (n = 12), MS (n = 12) and SLE (n = 12) LCL groups expressing the EBV encoded proteins as analyzed by flow cytometry. **Supporting Figure S2:** A) Relative expression of EBV DNA copy number examined by RT‐qPCR normalized to DNA concentration of LCLs from the HC (n=12), MS (n=12), and SLE (n=12) cohorts. **Supporting Figure S3**: A) – C); Correlations of *EBNA2* and *BZLF1* gene expression in HC (A, n=12), MS (B, n=12), and SLE (C, n=12) LCL cohorts. **Supporting Figure S4**: Gating strategy for flow cytometry analysis. **Supporting Table S1**: LCL information from HC, MS, and SLE groups describing sex, age at collection of blood sample, age at diagnosis, and disease severity (EDSS for MS group and SLEDAI‐2K for SLE group). **Supporting Table S2**: Primer sequences used in RT‐qPCR for EBV gene expression interrogation. **Supporting Table S3:** Primer and probe sequences for determination of relative EBV DNA copy number. **Supporting Table S4**: Empirical P values calculated by permutation test for pair wise comparison of gene expression measurements**. Supporting Table S5**: Statistics used for plotting arc diagrams**.**

## Data Availability

The data underlying this article are available in the Supplementary Materials.
