# Supplementary material for: Epstein‐Barr Virus Transformed B Cells From Systemic Lupus Erythematosus and Multiple Sclerosis Patients Differ in EBV Lytic and Latency Marker Expression
Source: Immun Inflamm Dis. 2026 Mar 23;14(3):e70411. doi: 10.1002/iid3.70411 (PMC13097320; doi:10.1002/iid3.70411)
Supplement: Supplementary file 1 — Supporting Figure S1A: Protein expression level of the key EBV encoded proteins EBNA1 (FITC), EBNA2 (PE‐Cy5), LMP1 (APC), BZLF1 (PE) compared to the respective gene expression level for HC (n=12), MS (n=12) and SLE (n=12) LCL groups. Supporting Figure S1B: Frequency of HC (n = 12), MS (n = 12) and SLE (n = 12) LCL groups expressing the EBV encoded proteins as analyzed by flow cytometry. Supporting Figure S2: A) Relative expression of EBV DNA copy number examined by RT‐qPCR normalized to DNA concentration of LCLs from the HC (n=12), MS (n=12), and SLE (n=12) cohorts. Supporting Figure S3: A) – C); Correlations of EBNA2 and BZLF1 gene expression in HC (A, n=12), MS (B, n=12), and SLE (C, n=12) LCL cohorts. Supporting Figure S4: Gating strategy for flow cytometry analysis. Supporting Table S1: LCL information from HC, MS, and SLE groups describing sex, age at collection of blood sample, age at diagnosis, and disease severity (EDSS for MS group and SLEDAI‐2K for SLE group). Supporting Table S2: Primer sequences used in RT‐qPCR for EBV gene expression interrogation. Supporting Table S3: Primer and probe sequences for determination of relative EBV DNA copy number. Supporting Table S4: Empirical P values calculated by permutation test for pair wise comparison of gene expression measurements. Supporting Table S5: Statistics used for plotting arc diagrams. [file IID3-14-e70411-s001.docx]

**
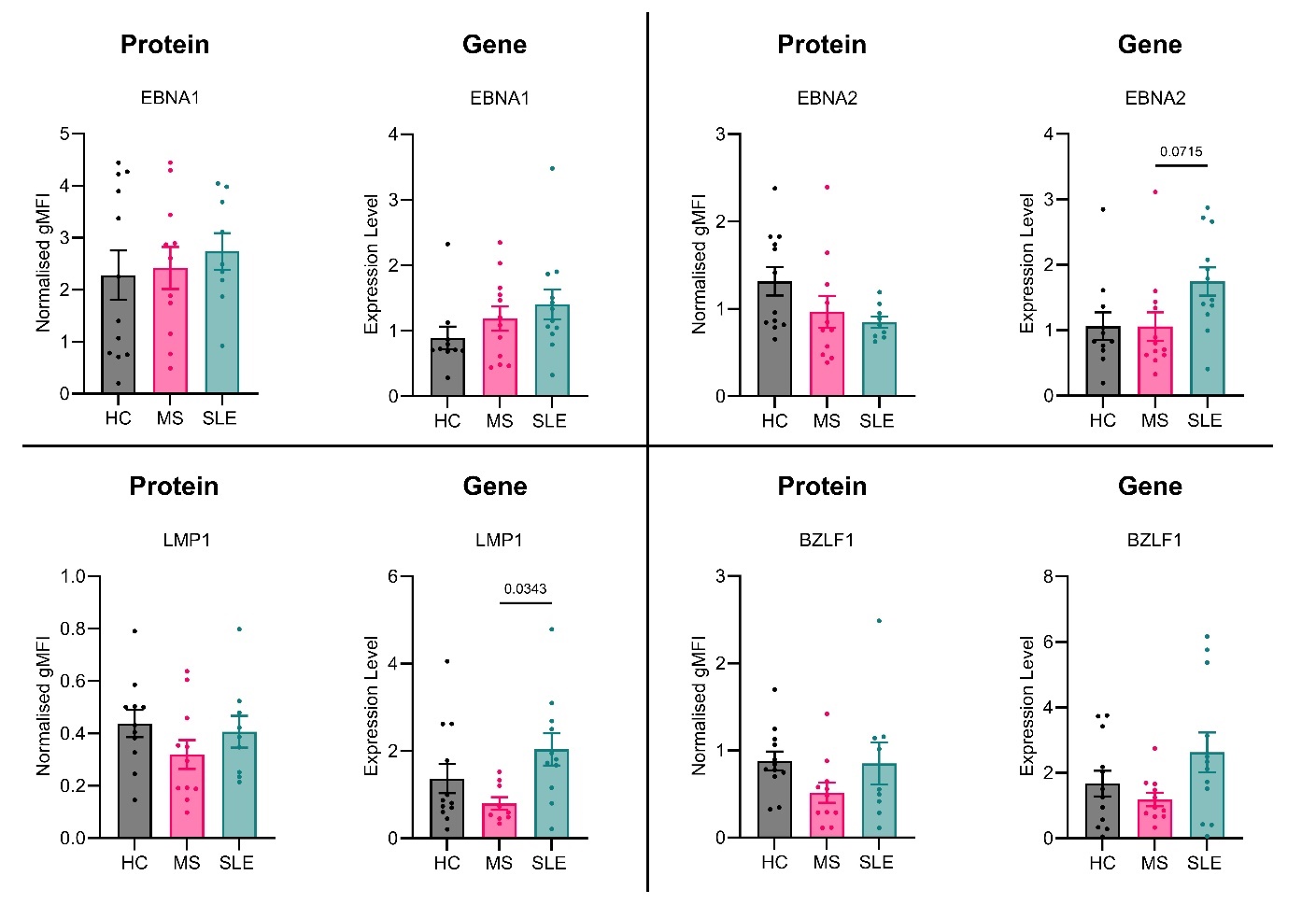
**

**Supplementary Figure 1A**: Protein expression level of the key EBV encoded proteins EBNA1 (FITC), EBNA2 (PE-Cy5), LMP1 (APC), BZLF1 (PE) compared to the respective gene expression level for HC (n=12), MS (n=12) and SLE (n=12) LCL groups. The mean expression of geometric mean fluorescence intensity (gMFI) is denoted by bars with individual data points superimposed and error bars representing ± standard error of the mean. The P value for pairwise comparisons are provided for statistically significant differences (p<0.05) and trends between groups.

**
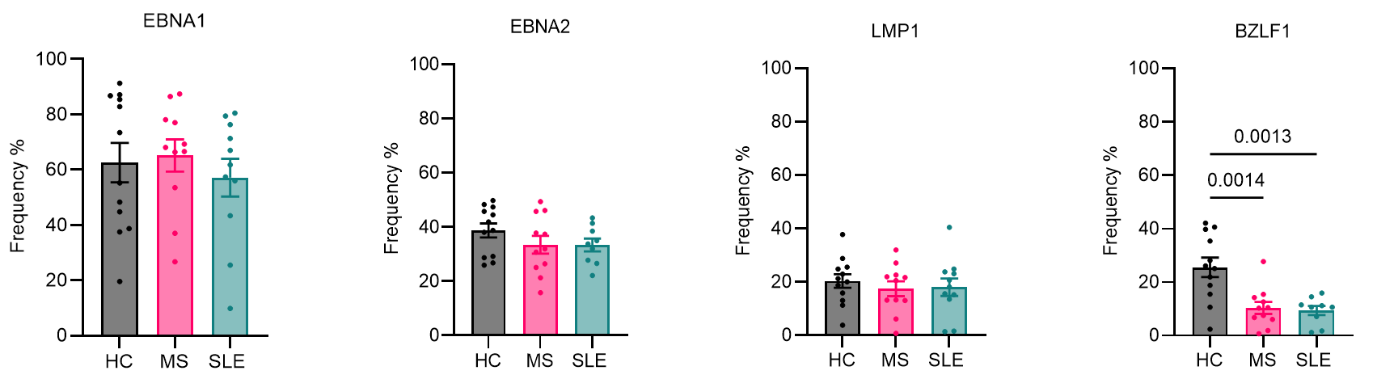
**

**Supplementary Figure 1B:** Frequency of HC (n=12), MS (n=12) and SLE (n=12) LCL groups expressing the EBV encoded proteins as analyzed by flow cytometry. Gating strategy to identify positive cells is illustrated in Supplementary Figure 4.


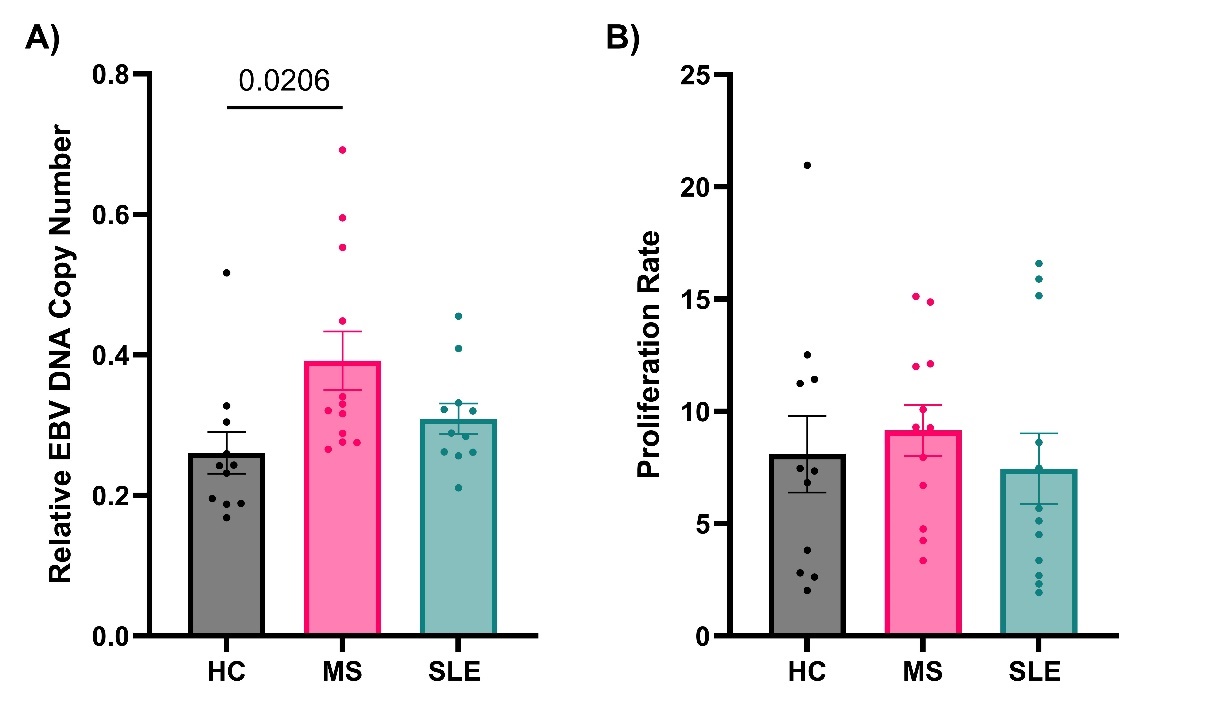
**Supplementary Figure 2:** A) Relative expression of EBV DNA copy number examined by RT-qPCR normalized to DNA concentration of LCLs from the HC (n=12), MS (n=12), and SLE (n=12) cohorts. B) Proliferation rate of LCLs from HC (n=12), MS (n=12) and SLE (n=12) donors determined by a fold change in cells between 0 hours and 120 hours. Mean EBV DNA copy number expression (A) or proliferation rate (B) are denoted by bars with individual data points superimposed and error bars representing ± standard error of the mean. The P value for pairwise comparisons are provided for statistically significant differences (p<0.05) between groups.


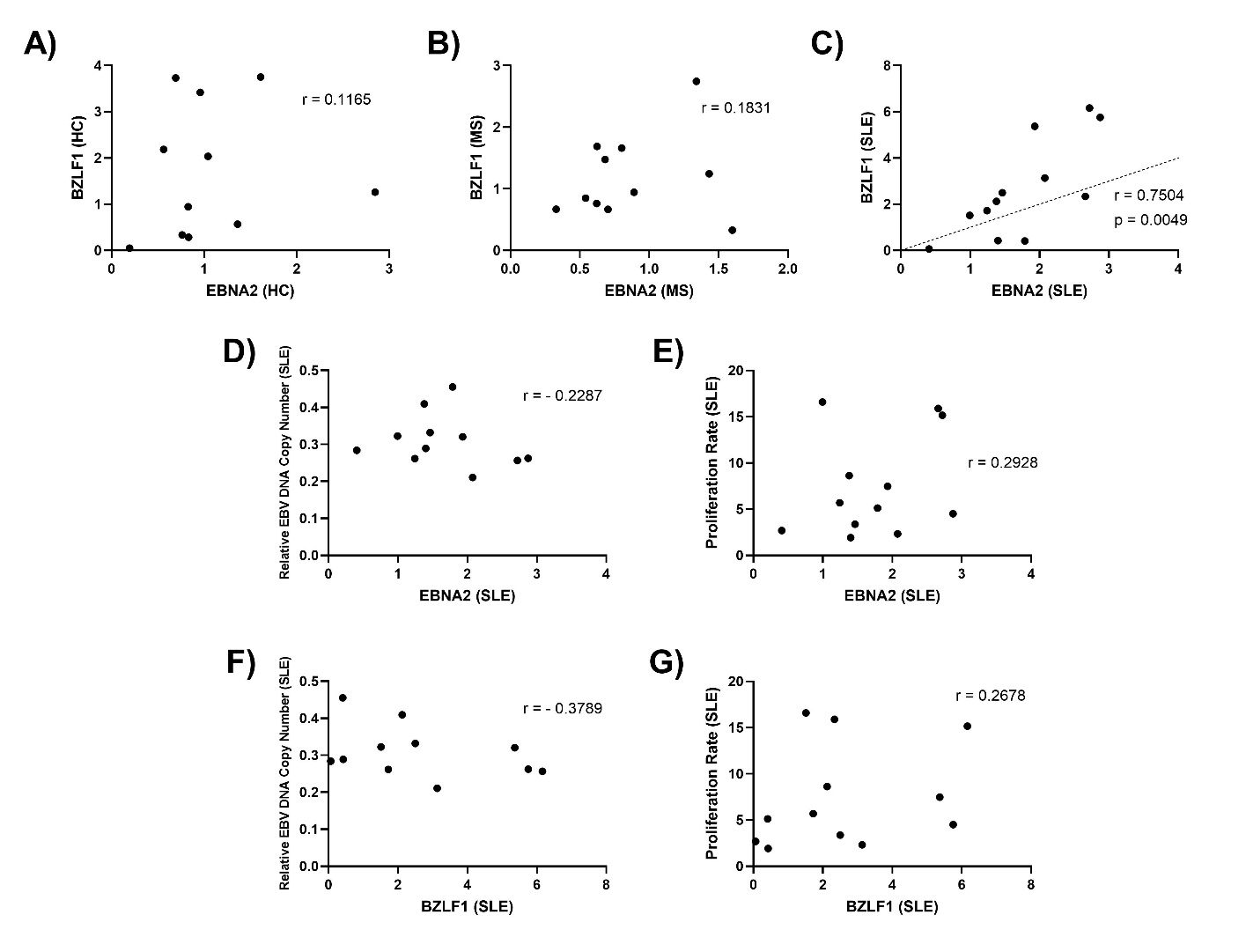


**Supplementary Figure 3**: A) – C); Correlations of *EBNA2* and *BZLF1* gene expression in HC (A, n=12), MS (B, n=12), and SLE (C, n=12) LCL cohorts. Pearson’s correlation coefficient is denoted by ‘r’. D) – E); Correlation of *EBNA2* gene expression in the SLE LCL group with relative EBV DNA copy number (D, n=12) and with proliferation rate (E, n=12). Pearson’s correlation coefficient is denoted by ‘r’. F) – G); Correlation of *BZLF1* gene expression in the SLE LCL group with relative EBV DNA copy number (F, n=12) and with proliferation rate (G, n=12). Pearson’s correlation coefficient is denoted by ‘r’. The P values are provided for statistically significant correlations.


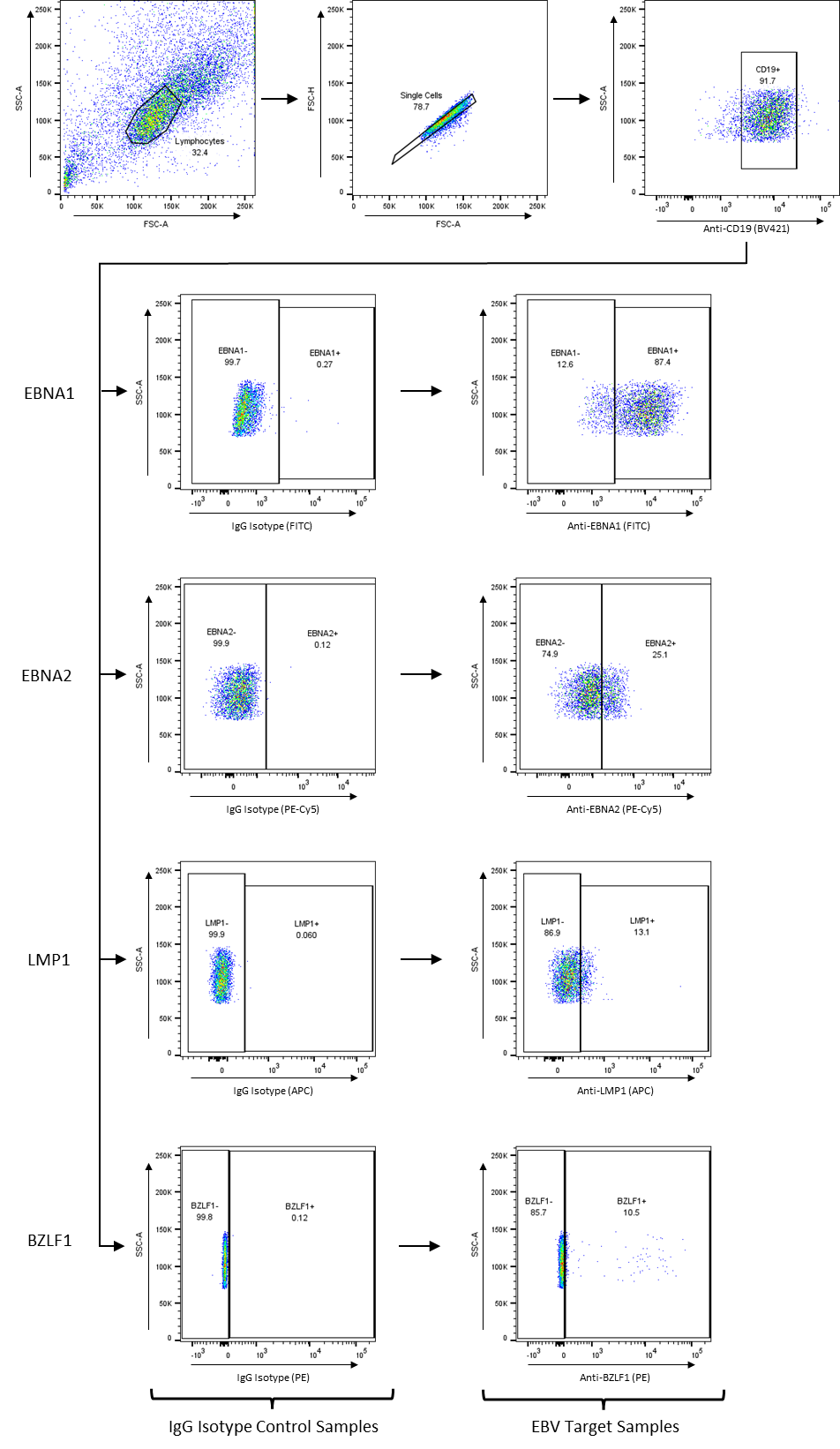


**Supplementary Figure 4**: Gating strategy for flow cytometry analysis. Firstly, cells of interest for both the isotype control samples and EBV target samples were gated based on morphology with subsequent gates for single cells and anti-CD19 positivity. From here, Isotype controls were used to determine positive and negative gates for EBNA1, EBNA2, LMP1 and BZLF1 expression.

**Supplementary Table 1**: LCL information from HC, MS, and SLE groups describing sex, age at collection of blood sample, age at diagnosis, and disease severity (EDSS for MS group and SLEDAI-2K for SLE group).

|  | Healthy Control | MS | SLE |
| --- | --- | --- | --- |
| Sex (F:M) | 9:3 | 9:3 | 9:3 |
| Age at Collection (Mean (SD)) | 40.5 (18.6) | 45.8 (11.6) | 40.7 (12.7) |
| Years After Diagnosis (Mean (SD)) | N/A | 15.8 (10.1) | 6.7 (5) |
| EDSS (Mean (SD)) | N/A | 3.9 (2) | N/A |
| SLEDAI-2K (Mean (SD)) | N/A | N/A | 4 (3.5) |

**Supplementary Table 2**: Primer sequences used in RT-qPCR for EBV gene expression interrogation.

| GENE | Size (bp) | Forward 5'-3' | Reverse 5'-3' |
| --- | --- | --- | --- |
| RPL30 | 107 | GCTGGAGTCGATCAACTCTAG | CCAATTTCGCTTTGCCTTGTC |
| EBNA1 | 521 | CCGCAGATGACCCAGGAGAA | TGGAAACCAGGGAGGCAAAT |
| EBNA2 | 129 | TTAGAGAGTGGCTGCTACGCATT | TCACAAATCACCTGGCTAAG |
| LMP1 | 210 | AATTTGCACGGACAGGCATT | AAGGCCAAAAGCTGCCAGAT |
| BZLF1 | 238 | TCTGAACTAGAAATAAAGCGATACAAGAA | TTGGGCACATCTGCTTCAACAGGA |
| BRLF1 | 115 | TGGCTTGGAAGACTTTCTGAGGCT | AATCTCCACACTCCCGGCTGTAAA |

**Supplementary Table 3:** Primer and probe sequences for determination of relative EBV DNA copy number.

| Target | Forward 5'-3' | Reverse 5'-3' | Probe |
| --- | --- | --- | --- |
| EBV DNA Copy Number | AAGGGCGCCAGCTTTTCT | ACTTTACAGACAGTGCACAGGAGAC | FAM-CCCCAGCCTGAGGC-Iowa BlackFQ |

**Supplementary Table 4**: Empirical P values calculated by permutation test for pair wise comparison of gene expression measurements**.**

| GENE | MS v HC | SLE v HC | SLE v MS |
| --- | --- | --- | --- |
| EBNA1 | 0.26316 | 0.09300 | 0.48080 |
| EBNA2 | 0.98333 | 0.03637 | 0.03596 |
| LMP1 | 0.18208 | 0.19806 | 0.00666 |
| BZLF1 | 0.29697 | 0.20069 | 0.04296 |
| BRLF1 | 0.21152 | 0.01797 | 0.00692 |

**Supplementary Table 5**: Statistics used for plotting arc diagrams**.**

| **Group** | **EBV marker one** | **EBV marker two** | **Pearson Correlation coefficient** | **P value** | **Sample size** | **Z prime** |  |  |
| --- | --- | --- | --- | --- | --- | --- | --- | --- |
|  |  |  |  |  |  |  |  | |
| HC | EBNA1 | EBNA2 | 0.93 | 3.68E-05 | 11 | 1.65 |  |  |
| HC | EBNA1 | LMP1 | 0.92 | 0.000128091 | 10 | 1.62 |  |  |
| HC | EBNA1 | BZLF1 | 0.30 | 0.396502501 | 10 | 0.31 |  |  |
| HC | EBNA1 | BRLF1 | 0.47 | 0.141063283 | 11 | 0.51 |  |  |
| HC | EBNA2 | LMP1 | 0.82 | 0.001988045 | 11 | 1.16 |  |  |
| HC | EBNA2 | BZLF1 | 0.09 | 0.791083431 | 11 | 0.09 |  |  |
| HC | EBNA2 | BRLF1 | 0.33 | 0.298727961 | 12 | 0.34 |  |  |
| HC | LMP1 | BZLF1 | 0.41 | 0.237862128 | 10 | 0.44 |  |  |
| HC | LMP1 | BRLF1 | 0.54 | 0.087694225 | 11 | 0.60 |  |  |
| HC | BZLF1 | BRLF1 | 0.88 | 0.000420563 | 11 | 1.35 |  |  |
| MS | EBNA1 | EBNA2 | 0.68 | 0.01502138 | 12 | 0.83 |  |  |
| MS | EBNA1 | LMP1 | 0.27 | 0.475600326 | 9 | 0.28 |  |  |
| MS | EBNA1 | BZLF1 | 0.41 | 0.210711485 | 11 | 0.44 |  |  |
| MS | EBNA1 | BRLF1 | 0.27 | 0.457039465 | 10 | 0.27 |  |  |
| MS | EBNA2 | LMP1 | -0.19 | 0.619230646 | 9 | -0.20 |  |  |
| MS | EBNA2 | BZLF1 | 0.18 | 0.590027093 | 11 | 0.19 |  |  |
| MS | EBNA2 | BRLF1 | 0.19 | 0.60313473 | 10 | 0.19 |  |  |
| MS | LMP1 | BZLF1 | -0.23 | 0.547441024 | 9 | -0.24 |  |  |
| MS | LMP1 | BRLF1 | -0.06 | 0.87510526 | 9 | -0.06 |  |  |
| MS | BZLF1 | BRLF1 | 0.71 | 0.021767558 | 10 | 0.88 |  |  |
| SLE | EBNA1 | EBNA2 | 0.39 | 0.204975499 | 12 | 0.42 |  |  |
| SLE | EBNA1 | LMP1 | 0.33 | 0.318575623 | 11 | 0.35 |  |  |
| SLE | EBNA1 | BZLF1 | 0.65 | 0.022983213 | 12 | 0.77 |  |  |
| SLE | EBNA1 | BRLF1 | 0.50 | 0.10143194 | 12 | 0.54 |  |  |
| SLE | EBNA2 | LMP1 | 0.16 | 0.639525705 | 11 | 0.16 |  |  |
| SLE | EBNA2 | BZLF1 | 0.75 | 0.004932598 | 12 | 0.97 |  |  |
| SLE | EBNA2 | BRLF1 | 0.60 | 0.040406642 | 12 | 0.69 |  |  |
| SLE | LMP1 | BZLF1 | 0.12 | 0.735161093 | 11 | 0.12 |  |  |
| SLE | LMP1 | BRLF1 | 0.37 | 0.26854964 | 11 | 0.38 |  |  |
| SLE | BZLF1 | BRLF1 | 0.77 | 0.003080122 | 12 | 1.03 |  |  |

**Supplementary Methods**

**RNA extraction and EBV gene expression**

Approximately 4 x 10^5^ cells were harvested from LCL cultures growing at a cell density of 5 x 10^5^ cells/ml and stored in 100 μl RLY lysis buffer at -80°C for future RNA extraction as previously described (2). RNA was purified from LCLs using the Bioline Isolate II RNA Mini Kit (Meridian Bioscience, Cat.# BIO-52072 ). cDNA was then synthesised using qScript cDNA SuperMix (Quanta Bio, Cat.# 95048) and stored at -20°C following a 1:5 dilution with Tris EDTA Buffer (Sigma Aldrich, Cat.# 93283).

For quantification of gene expression of the selected EBV life cycle markers, RT-qPCR was performed using Takara Master Mix (Takara Bio, Cat.# RR42LR) and the primer sequences listed in *Supplementary Table 2*. Assays were run in duplicate on a BioRad CFX Opus 96 qPCR system with the following protocol: 95°C for 10 minutes, 5 cycles of 95°C for 15 seconds, 64°C for 15 seconds, and 72°C for 30 seconds, 35 cycles of 95°C for 15 seconds, 60°C for 15 seconds, and 72°C for 30 seconds. A melt curve analysis was also performed to ensure that only a single product was obtained. *RPL30* was used as a housekeeping gene and expression levels of EBV-encoded genes were calculated using the 2^-ΔΔCT^ method as previously described (3).

**Flow Cytometry for EBV Protein Expression** - For the examination of EBV protein abundance, 4x10^5^ cells were collected from LCL cultures growing at a cell density of 5 x 10^5^ cells/ml, the same cohort explored for gene expression. After washing and blocking, cells were stained with CD19 (BV421 – BD Biosciences, Cat.# 562440). Staining for EBV markers was achieved using the BD Pharmingen Transcription Factor Buffer Set (BD Biosciences, Cat.# 562574) as directed. EBV markers include: anti-EBNA1 (Abcam, Cat.# ab8329) – FITC (Abcam, Cat.# ab102884), anti-EBNA2 (Abcam, Cat.# ab90543) – PE-Cy5 (Abcam, Cat.# ab102893), anti-LMP1 (Invitrogen, Cat.# MA5-33322) – APC (Abcam, Cat.# ab201807), anti-BZLF1 – PE (Santa Cruz Biotechnology, Cat.# sc-53904 PE), and corresponding isotype controls (IgG1 – BioLegend). EBNA1, EBNA2, and LMP1 antibodies were conjugated to their respective fluorochromes as directed by manufacturer’s instructions. Samples were acquired using the BD FACSCanto II (Becton Dickinson). Data was analysed using Flow Jo v10.10.0 (Becton Dickinson) and GraphPad Prism v10.2.3.

**EBV DNA Copy Number Quantification** - Approximately 1 x 10^6^ cells were harvested from LCL cultures growing at a cell density of 5 x 10^5^ cells/ml at the beginning of the proliferation rate experiment and stored in lysis buffer at -80°C as previously described (2). DNA was purified using the Bioline Isolate II Genomic DNA Kit (Meridian Bioscience, Cat.# BIO-52066). For quantification of EBV DNA copy number, RT-qPCR was performed using TaqMan Universal Master Mix II (Applied Biosystems. Thermo Fisher Scientific) with the primer sequences listed (*Supplementary Table 3*). Assays were run in duplicate on a BioRad CFX Opus 96 qPCR system. Ct values obtained were normalised by DNA concentration as measured by the Nanodrop 2000 Spectrophotometer.

**Proliferation Rate Assay** - Once the LCL cultures reached a cell density of 5 x 10^5^ cells/ml, cells were harvested and counted using the CytoFLEX (Beckman Coulter). For each LCL, 1 x 10^4^ cells were seeded in duplicate for 6 time points (0 hour and hours 72-168) in a U-bottom 96 well plate using fresh RPMI-1640 medium with 10% FBS. At each timepoint, cells were extracted, and the wells were washed with DPBS. Cell quantity was determined using the CytoFLEX and live cell counts were obtained using FlowJo (Becton Dickinson). Proliferation rate was determined by calculating the fold change between 0 hours and 120 hours.

**Statistical Analysis**

First, the ROUT method (Q = 1%) was applied to identify and remove outliers. Normality of gene expression data was assessed using the Anderson-Darling test with a p-value threshold of 0.01. If this threshold was passed, ANOVA and post-hoc Tukey HSD tests were used *(LMP1, BRLF1, BZLF1*), otherwise a Kruskal-Wallis test with Dunn’s multiple comparisons test was used (*EBNA1, EBNA2*). The analyses were undertaken using GraphPad Prism v10.2.3. To further assess the robustness of the observed differences, empirical p-values were estimated for each of the pairwise comparisons using a permutation test comprising 100,000 permutations. Due to the limited sample size, individual values were plotted on all figures to enable visualization of variation. No adjustments were applied beyond permutation testing.

Next, the collective function of the EBV life cycle markers within each group was examined by employing a network correlation analysis on the gene expression measurements. Pairwise Pearson’s correlation tests were utilized to achieve this. To counteract the impact of varying sample sizes on our correlation analysis, the Pearson correlation coefficients were normalized and scaled to Z-prime values using Fisher's Z transformation (5).

**References**

1. Afrasiabi A, Parnell GP, Fewings N, Schibeci SD, Basuki MA, Chandramohan R, et al. Evidence from genome wide association studies implicates reduced control of Epstein-Barr virus infection in multiple sclerosis susceptibility. Genome Med. 2019;11(1):26.

2. Keane JT, Afrasiabi A, Schibeci SD, Swaminathan S, Parnell GP, Booth DR. The interaction of Epstein-Barr virus encoded transcription factor EBNA2 with multiple sclerosis risk loci is dependent on the risk genotype. EBioMedicine. 2021;71:103572.

3. Livak KJ, Schmittgen TD. Analysis of relative gene expression data using real-time quantitative PCR and the 2(-Delta Delta C(T)) Method. Methods. 2001;25(4):402-8.

4. Mandage R, Telford M, Rodríguez JA, Farré X, Layouni H, Marigorta UM, et al. Genetic factors affecting EBV copy number in lymphoblastoid cell lines derived from the 1000 Genome Project samples. PLoS One. 2017;12(6):e0179446.

5. Fisher RA. Frequency distribution of the values of the correlation coefficient in samples of an indefinitely large population. Biometrika.10(4):507-21.
